# Breast cancer recognition by electrical impedance tomography implemented with Gaussian relaxation-time distribution (EIT–GRTD)

**DOI:** 10.2478/joeb-2024-0011

**Published:** 2024-08-12

**Authors:** Galih Setyawan, Prima Asmara Sejati, Kiagus Aufa Ibrahim, Masahiro Takei

**Affiliations:** 1 Department of Mechanical Engineering, Graduate School of Science and Engineering, Chiba University, Chiba, Japan; 2 Department of Electrical Engineering and Informatics, Vocational College, Universitas Gadjah Mada, Yogyakarta, Indonesia

**Keywords:** Breast cancer, impedance, tomography, recognition, Gaussian Relaxation-Time Distribution

## Abstract

The comparison between breast cancer recognition by electrical impedance tomography implemented with Gaussian relaxation time distribution (EIT-GRTD) and conventional EIT has been conducted to evaluate the optimal frequency for cancer detection *f*^cancer^. The EIT-GRTD has two steps, which are 1) the determination of the *f*^cancer^ and 2) the refinement of breast reconstruction through time-constant enhancement. This paper employs two-dimensional numerical simulations by a finite element method (FEM) software to replicate the process of breast cancer recognition. The simulation is constructed based on two distinct electrical properties, which are conductivity *σ* and permitivitty *ε*, inherent to two major breast tissues: adipose tissues, and breast cancer tissues. In this case, the *σ* and *ε* of breast cancer *σ*^cancer^, *ε*^cancer^ are higher than adipose tissues *σ*^adipose^, *ε*^adipose^. The simulation results indicate that the most effective frequency for breast cancer detection based on EIT-GRTD is *f*^cancer^ = 56,234 Hz. Meanwhile, conventional EIT requires more processing to determine the *f*^cancer^ based on image results or spatial conductivity analysis. Quantitatively, both EIT-GRTD and conventional EIT can clearly show the position of the cancer in layers 1 and 2 for EIT-GRTD and only layer 1 for conventional EIT.

## Introduction

Breast cancer is characterized by uncontrolled abnormal cell growth in breast tissue [1]. This critical condition underscores the imperative for heightened awareness, efficacious treatments, and prompt recognition to enhance patient survival rates. Conventional technologies used in breast cancer recognition include mammography, ultrasound, and biopsy. Mammography uses X-rays to identify breast abnormalities [2], while ultrasound uses sound waves for internal imaging [3]. A biopsy involves taking a small sample of breast tissue for microscopic analysis to detect cancer cells. Despite their advantages, these devices have limitations, such as being less sensitive to dense breast tissue on mammography [4], having limitations in detecting small lesions on ultrasound, and their invasive nature increases the risk of discomfort on biopsy. Therefore, precise, non-invasive, and real-time breast cancer recognition technologies are needed.

Electrical Impedance Tomography (EIT) has emerged as an affordable, non-invasive [5], radiation-free, and real-time medical imaging solution [6][7], particularly applicable in the breast area. EIT aims to reconstruct the impedance distribution within an object based on electrical measurements taken at the object’s boundaries. Since the 1970s, EIT has been the subject of active research, yielding numerous valuable and inspiring findings [8]. Numerous studies have employed electrical impedance tomography (EIT) for breast cancer detection, employing various layer configurations, electrode quantities, and methodologies. Gutierrez et al. [9] utilized eight circular electrodes within a single layer. Gomes et al. [10] and Rao et al. [11] employed 16 circular electrodes in a single layer. Conversely, Zarafshani et al. [12] utilized 85 electrodes arranged in a planar array, whereas Hong Sunjoo et al. [13] employed 92 electrodes distributed across multiple circular layers. The exploration of EIT encompasses various methodologies to reveal the conductivity distribution within an object [14]. While EIT systems offer certain advantages, they also face limitations, especially in the optimal frequency determination for cancer detection *f*^cancer^, necessitating further investigation within conventional EIT.

**Fig. 1:**
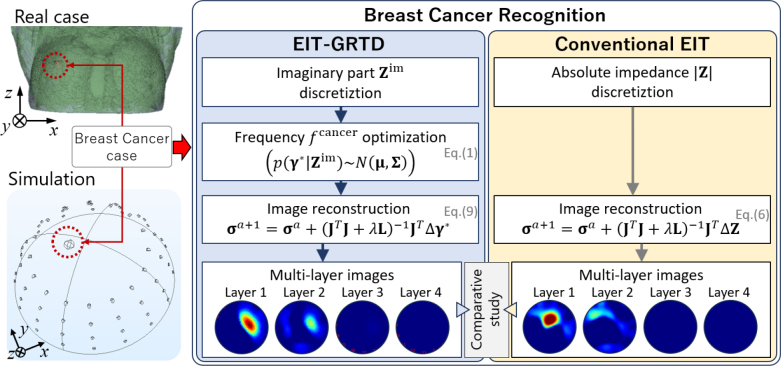
Overview of breast cancer recognition by EIT-GRTD versus conventional EIT

To overcome the drawbacks, integrating EIT with the Gaussian relaxation time distribution (GRTD) method could present a promising approach. This integration is anticipated to mitigate this limitation by simplifying the determination of optimal frequencies for cancer detection. This study has two objectives, which are 1) to conduct a comprehensive analysis of the *f*^cancer^ between EIT-GRTD and conventional EIT and 2) to perform a detailed comparison effectiveness of image reconstruction between EIT-GRTD and conventional EIT. **Fig. 1** shows the overview of the breast recognition by EIT-GRTD and conventional EIT.

## Breast cancer recognition by electrical impedance tomography implemented with Gaussian relaxation-time distribution (EIT-GRTD)

**Fig.1** shows the overview of breast cancer recognition by electrical impedance tomography with Gaussian relaxation-time distribution EIT-GRTD versus conventional EIT. The main difference between the two methods lies in determining the optimal frequency value *f*^cancer^ for breast cancer detection and in the reconstruction of the input image. EIT-GRTD utilizes the difference of the predicted distribution relaxation time function Δγ^*^ input, while conventional EIT rely on the discrepancy of impedance Δ**Z** between two measured voltages.

## Gaussian relaxation-time distribution (GRTD)

GRTD is a refinement of the conventional relaxation time distribution (RTD) using Gaussian distribution. With this approach, we can more accurately predict the relaxation-time distribution function $\gamma^{*}={\left(\gamma_{1}^{*},\ldots ,\gamma_{k}^{*},\ldots ,\gamma_{K}^{*}\right)}^{T}\in R^{K}$ from the imaginary part of impedance $Z^{\text{im}}={\left(Z_{1}^{\text{im}},\ldots ,Z_{m}^{\text{im}},\ldots ,Z_{M}^{\text{im}}\right)}^{T}\in R^{M}$. The correlation of imaginary impedance at m-*th* $\left(Z_{m}^{\text{im}}\right)$ and relaxation time distribution function at k-*th* $\left(\gamma_{k}^{*}\right)$ is depicted in the following equation [15]:

1 $$Z_{m}^{\text{im}}=-\overset{\infty }{\underset{-\infty }{\int }}\frac{2\pi f\tau \gamma_{k}^{*}}{1+{\left(2\pi f\tau \right)}^{2}}\text{d}\tau$$

where 2*πf* and *τ* are angular frequency within the measured spectrum and relaxation-times, respectively.

The joint distribution between **Z**^im^ and γ^*^ is expressed as follows [15]:

2 $$\left(p\left(\gamma^{*}\text{|}Z^{\text{im}}\right)\sim N\left(\mu ,\Sigma \right)\right)$$

where *N* is the Gaussian distribution, *p* is posterior distribution, **μ** and **Σ** are mean and covariance, respectively. The optimal frequency for detection the cancer is shown as follows:


3 $$f^{\text{cancer}}=\arg\;\max\left(p\left(\gamma^{*}\text{|}Z^{\text{im}}\right)\sim N\left(\mu ,\Sigma \right)\right)$$

The arg max function in eq.2 serves the purpose of determining the optimal value for the input variable that maximizes the objective function. Within this framework, the objective function pertains to the conditional probability in eq.2, wherein γ^*^ denotes the variable subject to optimization in relation to the dataset **Z**^im^. The γ^*^ term is computed across a range of frequencies at each measurement number, with the optimized frequency for each measurement determined by the highest γ^*^ peak. The overall optimized frequency is then selected based on the highest γ^*^ peak from the measurement number that best represents the object of interest.

## Breast Cancer Imaging

### Conventional EIT by frequency difference EIT

The conductivity distribution σ of breast cancer is expressed as [16]

4 $$\sigma ={\left[\sigma_{1}\left(r_{1}\right),\ldots \sigma_{g}\left(r_{g}\right),\ldots ,\sigma_{G}\left(r_{G}\right)\right]}^{T}\in {\mathbb{R}}^{G}$$

where $r_{g}=\left(x_{g},y_{g}\right)\in \mathbb{R}$ is the row vector at the mesh point (1 ≤ *g* ≤ *G*). Standard Jacobian **J** is utilized to obtain the **σ,** which consist of all combinations of the injection current *I* and measured impedance *Z*. The standard Jacobian is defined by

5 $$J=\left[{\text{J}}_{m}^{g},\ldots ,{\text{J}}_{m}^{g},\ldots {\text{J}}_{M}^{G}\right]\in {\mathbb{R}}^{M\times G}$$

where *G* is the total mesh number of spatial resolution and *M* is the total measurement number. The calculation of the ${\text{J}}_{m}^{g}$ for the *m*-th measured pattern at *g*-th mesh element is calculated by [17]

6 $$J_{mn}=\frac{\partial Z_{m}}{\partial \sigma_{G}}=-\int_{\text{Ω}}\nabla \phi \left(I^{a}\right)\cdot \nabla \phi \left(I^{b}\right)d\text{Ω}$$

Here, *ϕ*(*I^a^*) represents the potential field influenced by the injected current *I* into the *a*-th electrode, while *ϕ*(*I^b^*) denotes the potential field observed at the *b*-th measuring electrode. The variable *Z_m_* corresponds to the measured impedance at the *m*-th measurement instance (1≤*m*≤*M*), which is obtained from the measurement voltage *v_m_*. *σ_g_* represents the conductivity at the *g*-th mesh element (1≤*g*≤*G*). Ω represents the region of the electric field within the EIT sensor. Furthermore, Gaussian-Newton is utilized to obtain the conductivity distribution which is defined by [18]

7 $$\sigma^{a+1}=\sigma^{a}+{\left(J^{T}J+\lambda L\right)}^{-1}J^{T}\text{Δ}Z$$

where, *a*, *λ* and **L** are the a-*th* electrode, the regularization factors which is automatically obtained from L-curve [19] and regularization factors based on Tikhonov regularization, which is an identity matrix, respectively.

The $\text{Δ}Z={\left[\text{Δ}Z_{1},\ldots ,\text{Δ}Z_{\text{m}},\ldots ,\text{Δ}Z_{\text{M}}\right]}^{T}\in {\mathbb{R}}^{M}$ signifies the discrepancy in impedance between two measured voltage values: $Z^{f_{2}}$ is derived from injecting a high-frequency current (*f*_2_), and $Z^{f_{1}}$ is obtained from injecting a low-frequency current (*f*_1_) [20][21]. The Δ*Z*_m_ is expressed by

8 $$\text{Δ}Z_{m}=\frac{z_{m}^{f_{i}}-z_{m}^{f_{0}}}{z_{m}^{f_{0}}}$$

where the chosen fixed frequency is, *f*_0_= 1,000 Hz while the range of *f_i_* = 17,783 ~ 316,228 Hz, to reconstruct the conductivity **σ**.

In order to evaluate the performance of conventional EIT in breast recognition, the spatial mean conductivity is utilized using the following equation [22]

9 $$\sigma =\left(\sum_{g=1}^{g=G}\sigma_{g}\right)\text{/G}$$

where *σ_g_* denotes the conductivity distribution in the *g*-th mesh voxel.

### Time-constant enhancement by EIT-GRTD

EIT-GRTD, a Gauss-Newton is utilized to time constant enhance imaging which is expressed by [16] [19]

10 $$\sigma^{a+1}=\sigma^{a}+{\left(J^{T}J+\lambda L\right)}^{-1}J^{T}\text{Δ}\gamma^{*}$$

where Δγ^*^ is the difference of the predicted distribution relaxation time function at the optimal frequency *f*^opt^ expressed by

11 $$\text{Δ}\gamma^{*}={\left[\text{Δ}\gamma_{1}^{*},\ldots ,\text{Δ}\gamma_{m}^{*},\ldots ,\text{Δ}\gamma_{M}^{*}\right]}^{T}\in {\mathbb{R}}^{M}$$

where

12 $$\text{Δ}\gamma_{m}^{*}=\gamma_{m}^{*}\left(s\right)-\gamma_{m}^{*}\left(0\right)$$

where, $\gamma_{m}^{*}\left(s\right)$ and $\gamma_{m}^{*}\left(0\right)$ are inclusion and initial measurement distribution of relaxation time function, respectively. In this study, the $\gamma_{m}^{*}\left(0\right)$ was chosen based on the measurement results of the lowest relaxation time distribution value.

### Ethical approval

The conducted research is not related to either human or animal use.

## Simulation

### Simulation Setup and Condition

**Fig. 2:**
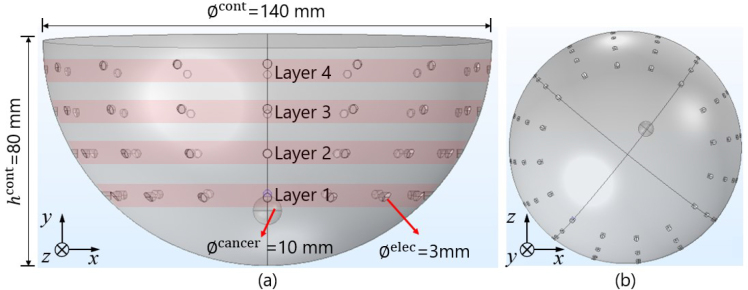
Simulation setup of breast cancer recognition in the case of (a) *z*-axis view, and (b) *y*-axis view

**Fig. 2** shows simulation setup of breast cancer recognition in the case of (a) z-axis view, and (b) y-axis view. In this study, the simulation is carried out by a finite element method (FEM) simulation software in 3D coordinates. In order to mimic breast cancer recognition, a breast container was designed in a half-spherical cup shape with diameter ∅^cont^ = 140 mm, a height *h*^cont^= 80 mm, and electrode diameter ∅^elec^= 3 mm.

**Fig. 3:**
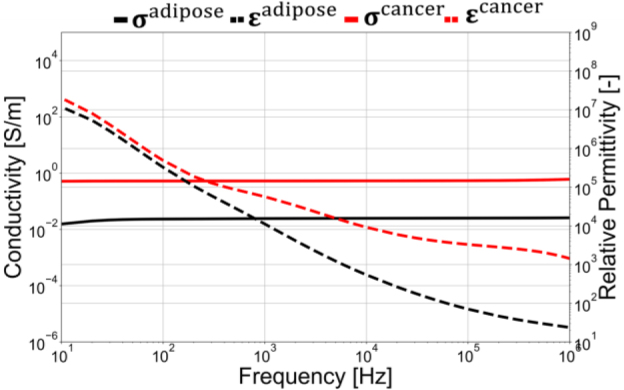
Electrical properties utilized in the simulation [24].

This study represents a preliminary investigation into the application of electrical impedance tomography (EIT) for breast cancer recognition. The use of simplified, hemispherical models in the simulation was chosen initially to establish foundational insights into impedance response, specifically focusing on impedance as a parameter for locating potential cancerous lesions within breast tissue. While we acknowledge that real breasts are soft and exhibit non-hemispherical shapes, these factors were simplified in this preliminary phase to facilitate controlled exploration of impedance characteristics. Spherical breast cancer is made with a diameter ∅^cancer^= 10 mm with the cancer position set close to layer 1. The breast container is composed of adipose tissue and breast cancer. **Fig. 3** shows the electrical properties utilized in this simulation. The electrical properties of breast cancer are employed with the electrical properties of breast glandular. In this case, we assumed that the conductivity *σ* and permittivity *ε* of breast cancer *σ*^cancer^, *ε*^cancer^ are higher than adipose tissues *σ*^adipose^, *ε*^adipose^. The authors employed glandular breast tissue conductivity and permittivity as representative parameters for breast cancer tissue, given the absence of specific data on its electrical properties along with the frequency data we used. Imaging cancer in dense breast tissue, which contains a substantial glandular component, is inherently challenging. However, data limitations led to this approach. Incorporating breast cancer-specific parameters into a background with a larger glandular component would enhance the simulation’s relevance and accuracy. Until breast cancer tissue data is available, glandular tissue attributes are utilized to approximate the situation as closely as possible.

### Simulation method

**Fig. 4:**
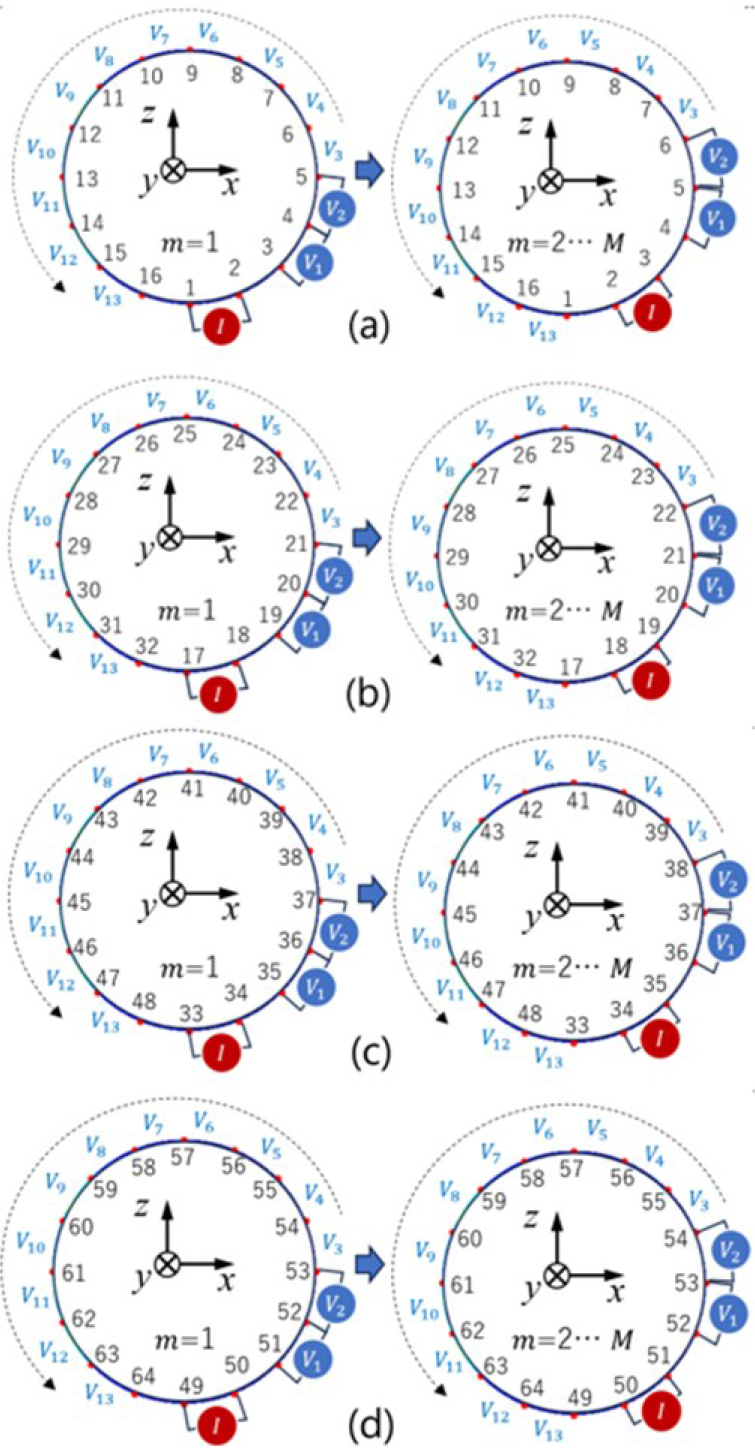
Adjacent pattern in simulation [25]

The simulation is carried out in this section with Multiphysics 3D simulation software. The simulation utilizes a breast container model of four layers with 64 electrodes. **Fig. 4** shows the adjacent pattern utilized in this stimulation with 1 mA injection current to acquire the voltage measurement *v_m_* at the *m*-th measurement. Measurements were performed with 6 frequency variations from 17,783 to 316,228 Hz with 1000 Hz as background. The Maxwell equation was applied to the simulation, defined by

13 $$\nabla .\left(\sigma \nabla \emptyset \right)+\nabla .\left(\varepsilon \nabla \left(\frac{\partial \emptyset }{\partial t}\right)\right)=0$$

where ∇, *σ*, ∅, and *ε* stand for gradient operator, conductivity [S/m], potential [V], and permittivity [F/m], respectively [14]. The simulation is assumed to be conducted under static conditions. In the boundary condition, the equation is assigned as follows [23]:

14 $$\nabla .\left(\sigma \nabla \emptyset \right)=0\;\text{on}\;\partial {\text{Ω}}^{breast}$$

to simplify, (*σ*∇∅) was replaced with **H**= (*σ*∇∅), so the boundary conditions are the following

15 $$\int_{\partial {\text{Ω}}^{e}HC}n.H\text{ds}=I_{0}\;\text{on}\;\partial {\text{Ω}}^{e}HC$$

16 $$\int_{\partial {\text{Ω}}^{e}LC}n.H\text{ds}=-I_{0}\;\text{on}\;\partial {\text{Ω}}^{e}LC$$

17 $$\begin{array}{cc} \underset{\partial {\text{Ω}}^{e}}{\int }n.H\text{ds}=0\;\text{on} & \partial {\text{Ω}}^{e}\text{/}\partial {\text{Ω}}^{e}HC,LC \end{array}$$

where **n** denotes the outward normal vector of the boundary, *∂*Ω^*e*^ represent the boundary of the electrode, *I*_0_ is current injection, and ds is the surface element of the electrode’s boundaries.

### Simulation Results

**Fig. 5:**
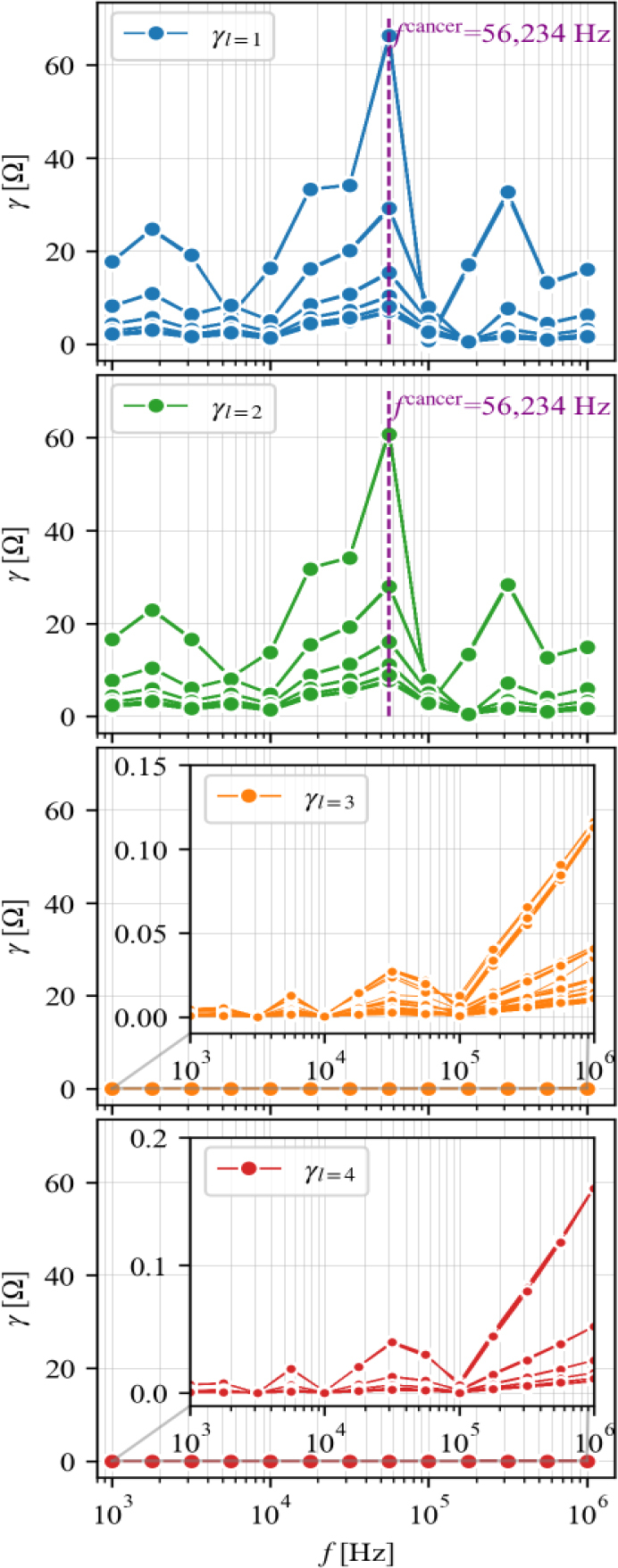
The determination of the frequency for cancer detection *f*^cancer^ in the simulation study

**Fig. 5** shows the results of frequency determination for cancer detection using the EIT-GRTD. The majority of *γ* peaks and dominance are observed at a frequency of 56,234 Hz in layers 1 and 2. However, in layers 3 and 4, EIT-GRTD does not identify a significant peak frequency compared in layer 1 and 2. EIT-GRTD only uses one frequency, whereas conventional EIT uses frequencies from 17,783 to 316,228 Hz to determine which is more appropriate for detecting cancer.

**Fig. 6:**
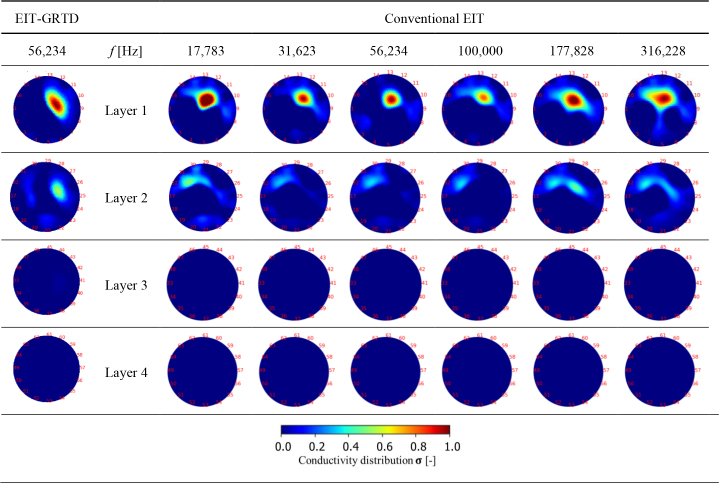
Image reconstruction result by time constant enhancement breast imaging at *f*^cancer^ = 56,234 Hz and conventional EIT

**Fig. 6** shows the comparison image results between the EIT-GRTD and conventional EIT. EIT GRTD and conventional EIT revealed successful cancer recognition in the first layer within the simulation environment at a distance of 5 mm from the center of the cancerous sphere to this layer. However, recognition accuracy declined for conventional EIT. It remained unchanged for EIT-GRTD when the cancerous sphere was placed farther away, such as in layer 2, which is positioned 20 mm from the cancerous sphere. Cancer was not visible in conventional EIT at layer 2, indicating limitations in the recognition system at greater distances. Only subtle indications of cancer could be observed through color gradients. Furthermore, in layers 3 and 4, positioned 35 mm and 50 mm from the center of the cancerous sphere, both EIT-GRTD and conventional EIT failed to detect cancer. This indicates more pronounced limitations in recognition capability as the distance between the sensors and the target increases.

## Discussion

Regarding the location of the cancer detection position shown in **Fig. 6**, there are significant differences between the EIT-GRTD method and conventional EIT. In both methods, cancer can be detected accurately in the same position in layer 1 compared to simulation setting. However, when switching to layer 2, the EIT-GRTD method is still able to identify the position of cancer precisely, while in conventional EIT, the cancer detection position is not clearly visible. Both methods exhibited noise on Layer 2. Although the object was isolated to Layer 1 in the simulated environment, noise appears in Layer 2 for both the proposed method and the conventional EIT method. This indicates the need for a noise-filtering method in future research to improve accuracy. Notably, in the proposed method, the noise in Layer 2 is not random but instead reveals the location of the object in Layer 1. In contrast, the noise in the conventional method is random and does not provide useful information about the lesion’s location. This distinction suggests that, while noise filtering is necessary, the noise characteristics in the proposed method still offer valuable insights into the object’s position.

**Fig. 7:**
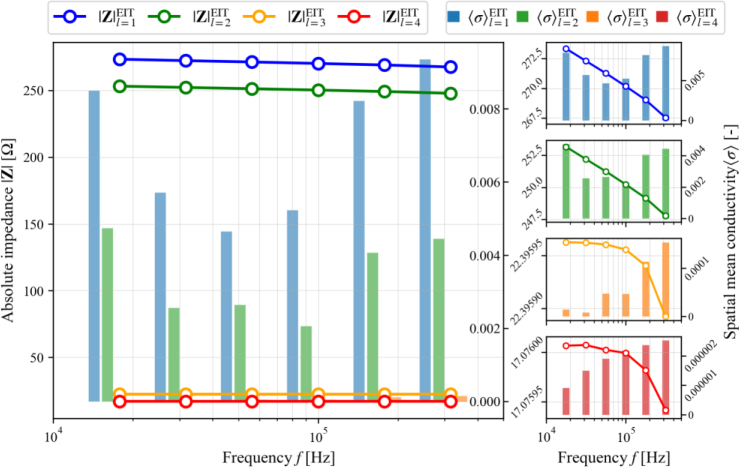
Absolute impedance |**Z**| and spatial mean conductivity 〈*σ*〉 plot in all layers

The analysis of absolute impedance and spatial mean conductivity in four layers is also needed in conventional EIT to determine the optimal frequency of cancer detection. **Fig. 7** shows the absolute impedance and spatial mean conductivity in four layers of the breast container with increasing frequency. Layer 1 has a higher absolute impedance compared to the other layers. The absolute impedance value in this simulation decreases with increasing frequency. The spatial mean conductivity values exhibit a pattern similar to the absolute impedance, which indicates the highest value at Layer 1.

However, a different pattern is observed as the frequency increases. There is a decrease in the spatial mean conductivity values from 17,783 Hz to 56,234 Hz, which then rises again with the increase in frequency until reaching 316,228 Hz. On the other hand, layers 3 and 4 consistently show an increase in spatial average conductivity as frequency increases. This complex behavior highlights the intricate dynamics governing the impedance and conductivity responses within the container breast model as frequency changes. This analysis still faces challenges in determining the best frequency for detecting cancer. Overall, the comparison of reconstruction results between EIT-GRTD and conventional EIT shows the superiority of EIT-GRTD in determining the frequency of cancer detection. The image results from EIT-GRTD are also closer to the settings in the simulation.

For future research, a more comprehensive comparison of the two imaging approaches could be achieved by considering the impact of noise. Moreover, the inclusions used in this study were of a single size and position; therefore, future research should incorporate variations in size and position to better assess the EIT-GRTD’s reliability. Additionally, future studies need to include experimental validation to verify the simulation results and ensure that the proposed methods are effective in practical applications.

## Conclusion

The comparison between breast cancer recognition by electrical impedance tomography implemented with Gaussian relaxation time distribution (EIT-GRTD) and conventional EIT has been conducted to evaluate the optimal frequency for cancer detection *f*^cancer^. The results show that the EIT-GRTD is easier to determine the frequency for cancer detection at *f*^cancer^ = 56,234 Hz than conventional EIT. Quantitatively, EIT-GRTD rapidly determines the correct position of cancer compared to conventional EIT, showcasing its capability to accurately locate the cancerous site.
